# Study on the views and methods of ultrasonic screening and diagnosis for abnormal aortic arch in infants

**DOI:** 10.1186/s12947-021-00237-2

**Published:** 2021-01-14

**Authors:** Xinjian He, Jiaoyang Chen, Gaoyang Li

**Affiliations:** Department of Ultrasoud Diagnosis, Children’s Hospital of Hebei Province, Hebei Medical University, Shijiazhuang, China

**Keywords:** Echocardiography, An infant, Aortic arch anomalies, The three-vessel and tracheal view

## Abstract

**Background:**

The purpose of this study was to explore echocardiographic views and methods of aortic arch anomalies in infants, so as to improve the screening sensitivity and diagnostic accuracy.

**Methods:**

140 children with abnormal aortic arch diagnosed by ultrasound in Children’s Hospital of Hebei Province from January 2014 to December 2019 were selected for retrospective analysis. All were confirmed by surgery or/and computerized tomography angiography. Series of views for aortic arch (the three-vessel and tracheal view, aortic arch short axis view, left aortic arch long axis view, aortic arch long axis continuous scan views) were performed in all cases on the basis of the routine views of echocardiography. The screening sensitivity and diagnostic coincidence rate of different echocardiographic views for aortic arch anomalies were analyzed.

**Results:**

Among the 140 infants, right aortic arch were 21 cases (6/21 were accompanied by mirror branch and 15/21 were with aberrant left subclavian artery). Left aortic arch with aberrant right subclavian artery were 2 cases, and double aortic arch with both arches open were 20 cases. Double aortic arch with left arch atresia were 2 cases, and atresia of the proximal aorta with aortic arch dysplasia was 1 case. Coarctation of the aorta were 67 cases, and interruption of aortic arch were 27 cases. All the patients were correctly diagnosed except that 2 infants with interruption of aortic arch were incorrectly diagnosed as coarctation of the aorta, and 1 infant with coarctation of the aorta was misdiagnosed as interruption of aortic arch by echocardiography. The screening sensitivities of four views and four-view combination for abnormal aortic arch were 99.3, 73.6, 87.1, 99.3, and 100%; the diagnostic coincidence rates were 85.7, 27.1,66.4, 95.0%, and 97.9% respectively. On the basis of traditional left aortic long axis view, other three views had their own advantages. The screening sensitivity and diagnostic coincidence rate of four-view combination were significantly improved.

**Conclusions:**

The three-vessel trachea view is simple and feasible, which is suitable for screening abnormal aortic arch. The combination of four views conduces to improving screening sensitivity and diagnostic accuracy of aortic arch abnormalities.

## Background

Aortic arch anomalies are common congenital heart malformations, and ultrasonic diagnosis of such malformations is challenging. Failure to detect these diseases in time is the main cause of morbidity and mortality in severe cases of infants [1]. The three-vessel and tracheal view is one of the internationally recognized important views for prenatal ultrasonic diagnosis of fetal congenital heart diseases [2, 3]. This view was applied to infants in this study for the first time, combining with series of views of aortic arch to diagnose and analyse aortic arch anomalies in infants. The objective of this study is to explore appropriate echocardiographic views and methods of aortic arch anomalies in infants and to improve the screening sensitivity and diagnostic accuracy.

## Methods

### Object

140 children with abnormal aortic arch diagnosed by ultrasound in Children’s Hospital of Hebei Province from January 2014 to December 2019 were collected for retrospective analysis. All were proved by surgery or/and computerized tomography angiography (CTA). Eighty-two cases were male, and 58 cases were female. Their ages ranged from 7 h to 12 months. The median age was 72 days and the mean age was 117 days. One hundred twenty-six cases were accompanied with other cardiac malformations, including 4 with tetralogy of Fallot, 1 with single ventricle, 1 with pulmonary atresia, 1 with Berry syndrome, 1 with aortopulmonary septal defect. Other malformations were mainly atrial septal defect, ventricular septal defect and patent ductus arteriosus.

### Inclusion criteria

Available aortic arch views should be obtained by ultrasound among the infants who were diagnosed with aortic arch anomalies and all were proved by surgery or/and CTA.

### Exclusion criteria

Infants who could not obtain the available views of aortic arch or cases who were not determined by surgery or/and CTA should be excluded.

### Equipment

Philips IE 33 with probe S5–1 and S8–3.

### Methods of examination

The children took supine position or left-lateral position. When examining the suprasternal fossa, raise the shoulder and tilt the head and neck back. Appropriate sedation should be given to children if they did not cooperate. Firstly, follow the “cardiac segmental diagnostic method” for systematic ultrasonic examination, then a series of views for the aortic arch were performed (the three-vessel and tracheal view, aortic arch short axis view, left aortic arch long axis view and aortic arch long axis continuous scan views). The position of the trachea which is in front of esophagus was determined by esophageal imaging on the three-vessel and tracheal view by means of drinking water or moving nasogastric feeding tube. Then the type of vascular ring was diagnosed definitely according to the relationship between vessels and trachea.

**Fig. 1:**
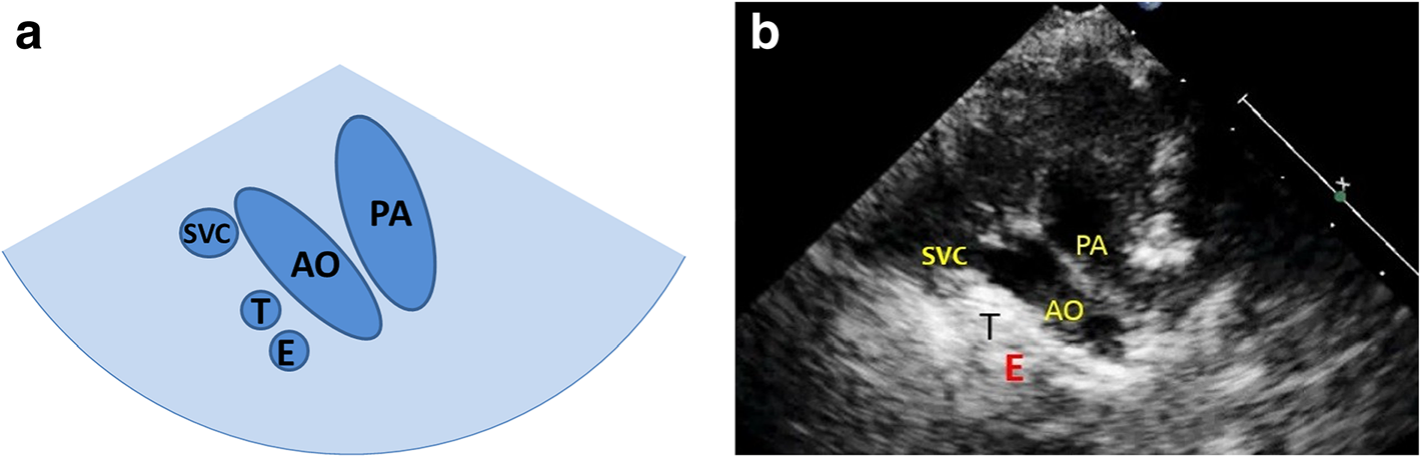
Normal three-vessel and tracheal view. **a** Schematic drawing. **b** Ultrasound images. The vena cava is located on the right side of the trachea. The pulmonary artery is connected to the descending aorta through the ductus arteriosus or ductal ligament, forming a symmetrical “V” sign with the aorta (the inner diameter of the aorta and the pulmonary artery is similar), both of which are located on the left side of the trachea. AO: aorta; E: esophagus; PA: pulmonary artery; T: trachea; SVC: superior venae cava

The three-vessel and tracheal view: firstly, we obtained the parasternal great vessels level short-axis high position view (similarly to fetal three-vessel view). On this basis, we made the acoustic beam skew toward the head and obtained the view which was similar to the fetal three-vessel and tracheal view. Then we could observe the position, number, morphological proportion. The position of the aortic arch is relative to the trachea. The aortic arch on the left side of the trachea is the left aortic arch (LAA), and aortic arch on the right side of the trachea is the right aortic arch (RAA). If the left and right sides of the trachea both have aortic arches, it is double aortic arch (DAA). Under normal circumstances, the aortic arch is located on the left side of the trachea, and sends out the brachiocephalic trunk, left common carotid artery, and left subclavian artery successively. The inner diameter of the infants’ aortic arch is similar to that of the pulmonary artery (close to 1:1), which appears as a nearly symmetrical V-shape on the three-vessel trachea view (Fig. 1).

**Fig. 2:**
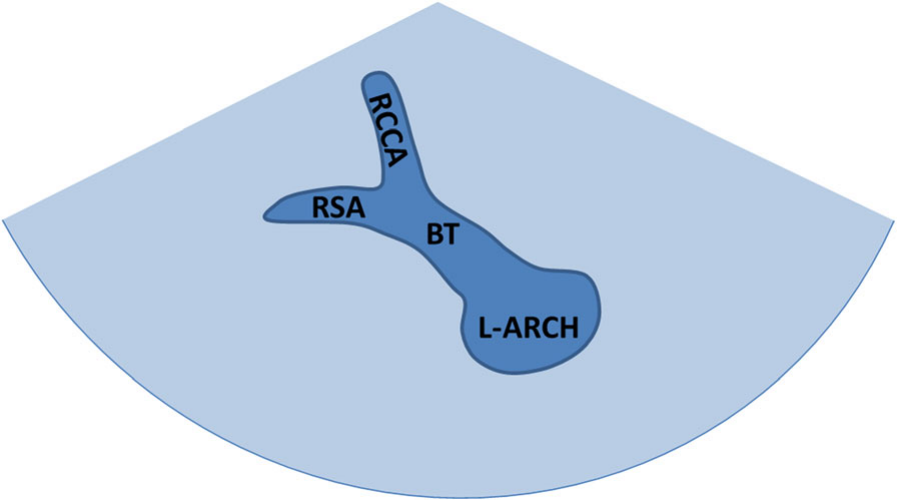
Schematic drawing of normal aortic arch short axis view. The normal aortic arch shows that the first branch points to the right anterior, and the proximal bifurcation can be seen (the right brachiocephalic trunk artery sends out the right common carotid artery and right subclavian artery). L-ARCH:left aortic arch; BT: brachiocephalic trunk; RCCA: right common carotid artery; RSA: right subclavian artery

The aortic arch short axis view: get the transverse scan of suprasternal fossa with probe maker pointing to the left, then we could observe the number, inner diameter, and first branch of the aortic arch (Fig. 2).

**Fig. 3:**
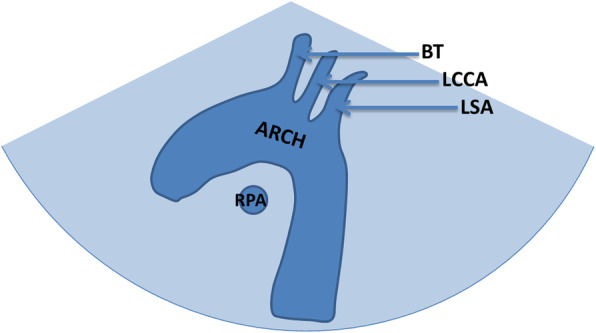
Schematic drawing of normal left aortic arch long axis view. **a** Schematic drawing. **b** Ultrasound images. The left aortic arch is continuous and sends out the brachiocephalic trunk and left common carotid artery and left subclavian artery, right pulmonary artery is under the aortic arch. ARCH:aortic arch; BT: brachiocephalic trunk; LCCA: left common carotid artery; LSA: left subclavian artery; RPA:right pulmonary artery

The left aortic arch long axis view: Try to make acoustic beam parallel with sternum of the infant with the probe marker pointing to the infant’s left shoulder, then we could observe the shape, inner diameter and continuity of aortic arch (Fig. 3).

The aortic arch long axis continuous scan views: firstly, contrarotated the probe until the probe marker pointed to infant’s right shoulder on the basis of left aortic arch long axis view, and tried to make acoustic beam parallel with sternum of an infant. Then we could obtain a series of sagittal views of aortic arch. We made the acoustic beam tilt gradually toward the head on the basis of aortic arch short axis view, and we could get a series of coronal views of aortic arch. Comprehensively judge the position, number, shape, branch and continuity of aortic arch by observing all images obtained during this operation.

We summarized the echocardiographic features of different types of aortic arch anomalies in each view, and compared ultrasonic diagnosis results with CTA or/and surgery. The screening sensitivity, diagnostic coincidence rate, advantages and disadvantages of different views of aortic arch anomalies were analyzed in order to acquire a reliable method of screening and diagnosis.

## Results

### Diagnostic results of different types of aortic arch anomalies on different views by echocardiography

One hundred forty infants with aortic arch anomalies were verified by CTA or/and surgery. RAA were 21 cases (6/21 were accompanied by mirror branch and 15/21 were with aberrant left subclavian artery (U - shaped vascular ring), and LAA with right aberrant subclavian artery (C - shaped vascular ring) were 2 cases. DAA with both arches open (O - shaped vascular ring) were 20 cases, and DAA with left arch atresia (similar O - shaped vascular ring) were 2 cases. Atresia of the proximal aorta with aortic arch dysplasia was 1 case. Sixty-seven cases were coarctation of the aorta (COA) and 27 cases were the interrution of the aortic arch (IAA). All the patients were correctly diagnosed except that 2 infants with interruption of aortic arch were incorrectly diagnosed as COA, and 1 infant with COA was misdiagnosed as interruption of aortic arch by echo. The screening sensitivities of three-vessel and tracheal view, aortic arch short axis view, left aortic arch long axis view, aortic arch continuous scan views and four-view combination for abnormal aortic arch were 99.3, 73.6, 87.1, 99.3, and 100% respectively; the diagnostic coincidence rates were 85.7, 27.1,66.4, 95.0%, and 97.9% respectively. Detailed diagnosis of various types of aortic arch anomalies on different views were shown in Tables 1, 2.

1. The three-vessel and tracheal view: the sensitivity of this view was high and the position, number, internal diameter ratio and continuity of aortic arch could be observed. Typical types of aortic arch anomalies in our group had characteristic images on this view (detailed descriptions were in paragraph 2 of this section). This view is easy to operate, and its advantage of visually showing the relationship between the vascular ring and the trachea is irreplaceable. However, this view had some limitations in showing the branches of the brachiocephalic artery.
2. The aortic arch short axis view: compared with the traditional left aortic arch long axis view, the short axis of the aortic arch increases the display of the DAA. According to the branch of the first brachiocephalic artery, we can infer the position of aortic arch, and whether there is aberrant subclavian artery. Speculative diagnosis were made correctly in cases with DAA, RAA and aberrant subclavian artery by this view. But abnormities could no be found easily on this view in some cases of COA of which the proximal aortic arch near the first branch were well developed.
3. The left aortic arch long axis view: The shape, internal diameter and continuity of the LAA can be shown clearly on this view, but DAA and RAA could not be observed directly because of scanning angle limitation.
4. The aortic arch long axis continuous scan views: these views compensated deficiencies of the left aortic arch long axis view on observation of DAA and RAA, and increased the detection rate of the aberrant subclavian artery significantly.

**Table 1:** Ultrasonic Screening and diagnosis of 140 cases with aortic arch abnormalities in each view and four-view combination

Classification	Number of cases	the three-vessel and tracheal view		the aortic arch short axis view		the left aortic arch long axis view		the aortic arch long axis continuous scan views		four-view combination	
		(−)	abnormity	(−)	abnormity	(−)	abnormity	(−)	abnormity	(−)	abnormity
Right aortic arch with aberrant left subclavian artery	15	0	15 (6△ + 9☆)	0	15 (15▲)	0	15 (15▼)	0	15 (12△ + 3☆)	0	15 (12△ + 3▲)
Right aortic arch with mirror branch	6	0	6 (6☆)	0	6 (6▲)	0	6 (6▼)	0	6 (6△)	0	6 (6△)
Left aortic arch with aberrant right subclavian artery	2	1☆	1 (1△)	0	2 (2▲)	1☆	1 (1△)	1☆	1 (1△)	0	2 (1△ + 1▲)
Double aortic arch with both arches open	20	0	20 (20△)	0	15 (15▲)	17☆	3 (3▼)	0	20 (20▲)	0	20 (20△)
Double aortic arch with left arch atresia	2	0	2 (2▲)	0	2 (2▼)	0	2 (2▼)	0	2 (2▲)	0	2 (2▲)
Aortic proximal atresia	1	0	1 (1▼)	0	1 (1▼)	0	1△	0	1△	0	1△
Coarctation of the aorta	67	0	67 (66△ + 1★)	37☆	30 (30▼)	0	67 (66△ + 1★)	0	67 (66△ + 1★)	0	67 (66△ + 1★)
Interruption of the aortic arch	27	0	27 (25△ + 2★)	0	27 (27▼)	0	27 (25△ + 2★)	0	27 (25△ + 2★)	0	27 (25△ + 2★)
Screening sensitivity			99.3%		73.6%		87.1%		99.3%		100.0%
Diagnostic coincidence rate			85.7%		27.1%		66.4%		95.0%		97.9%

△accurate diagnosis ▲speculative diagnosis ☆missed diagnosis ★incorrect diagnosis ▼This view does not show a normal image of the aortic arch, but this view alone cannot make a accurate diagnosis

**Table 2:** Ultrasonic screening and diagnosis of 140 cases with aortic arch abnormalities in the three-vessel and tracheal view

Classification	Num-ber of cases	the three-vessel and tracheal view					
		U-shaped vascular ring	C-shaped vascular ring	O-shaped vascular ring	similar O-shaped vascular ring	asymmetric “V” sign	“0 l” sign
Right aortic arch with aberrant left subclavian artery	15	15 (6△ + 9☆)	0	0	0	0	0
right aortic arch with mirror branch	6	6 (6☆)	0	0	0	0	0
Left aortic arch with aberrant right subclavian artery	2	0	1 (1△ + 1☆)	0	0	0	0
double aortic arch with both open arche	20	0	0	20 (20△)	0	0	0
Double aortic arch with left arch atresia	2	0	0	0	2 (2▲)	0	0
Aortic proximal atresia	1	0	0	0	0	1 (1▼)	0
Coarctation of aorta	67	0	0	0	0	66 (66△)	1 (1★)
Interruption of aortic arch	27	0	0	0	0	2 (2★)	25 (25△)

△accurate diagnosis ▲speculative diagnosis ☆missed diagnosis ★incorrect diagnosis ▼This view does not show a normal image of the aortic arch, but this view alone cannot make a accurate diagnosis

9☆ 9 cases were only diagnosed right aortic arch, but left subclavian artery aberration was not found

6☆ 6 cases were only diagnosed right aortic arch, but mirror branches was not found

1☆ 1 case were missed diagnosis of aberrant right subclavian artery

2▲ speculative diagnosis of double aortic arch with left aortic arch atresia in 2 cases

1▼ dysplasia of aortic arch was found in 1 case, and the blood flow of ductus arteriosus shunted from pulmonary artery to aortic arch, but definite diagnosis of atresia of aortic arch could not be made

1★ 1 case of coarctation of aortic arch was incorrectly diagnosed as interruption of aortic arch

2★ 2 cases of interruption of aortic arch (isthmus atresia) were incorrectly diagnosed as coarctation of aorta

### Echocardiographic features of various aortic arch anomalies on the three-vessel and tracheal view

**Fig. 4:**
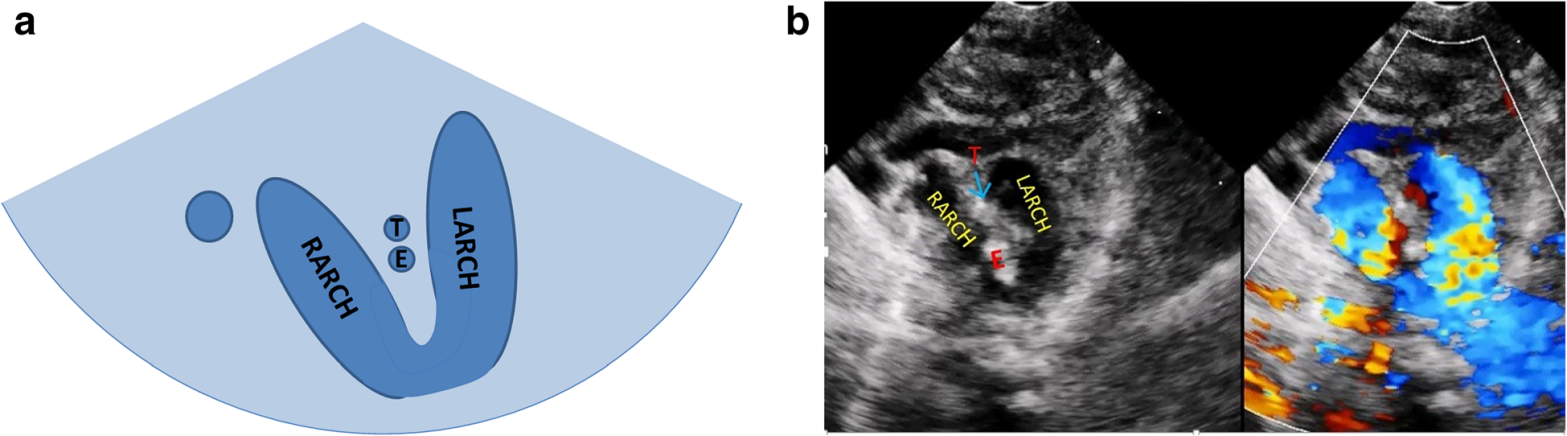
Double aortic arch with both arches open on three-vessel and tracheal view. **a** Schematic drawing. **b** Ultrasound images. The left and right aortic arch on both sides of the trachea ran backward and downward, and connected with the descending aorta, enclosing the trachea and esophagus to form an “O-shaped” vascular ring. E:esophagus; LARCH:left aortic arch; RARCH:right aortic arch; T:trachea

**Fig. 5:**
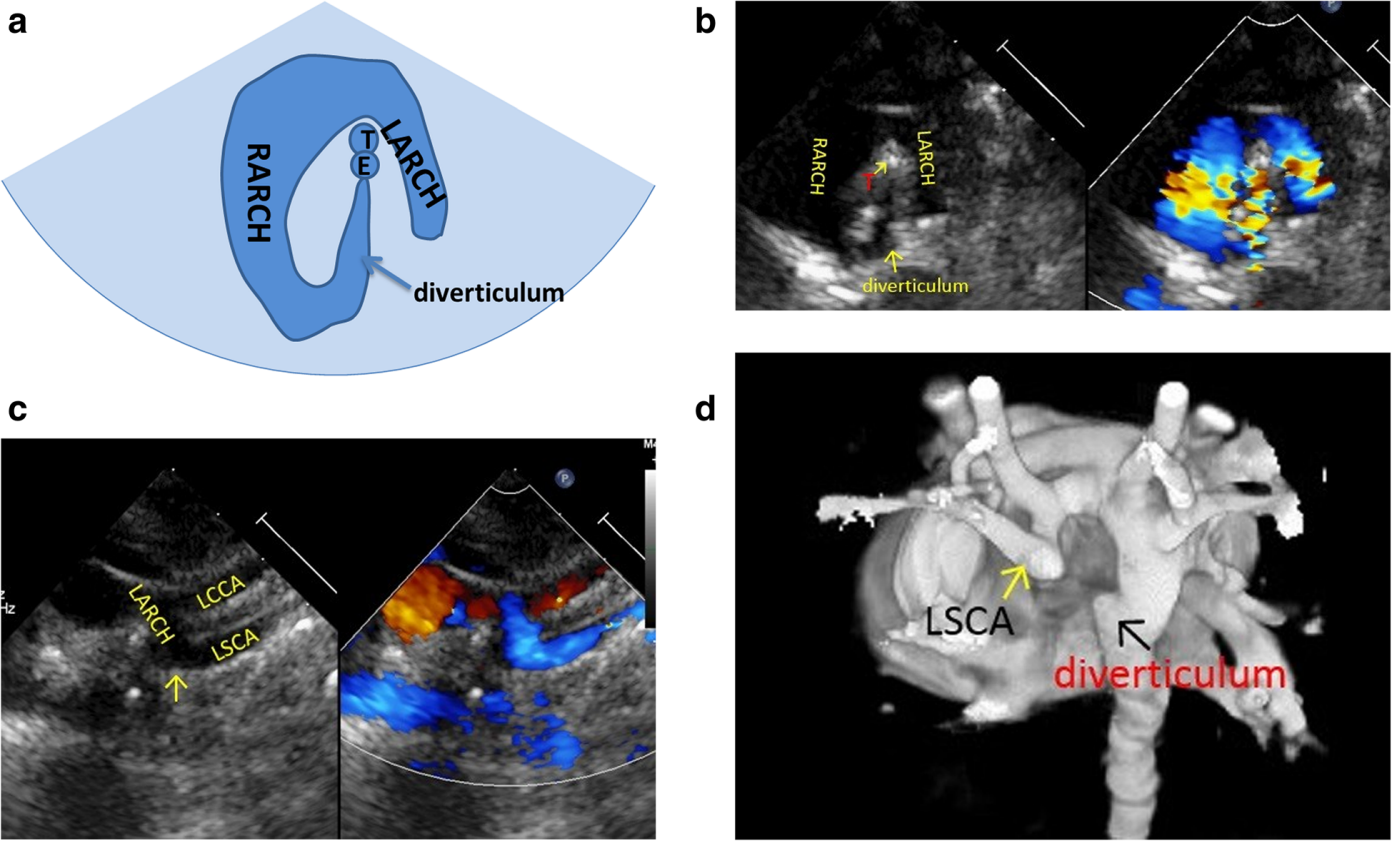
Double aortic arch with left arch atresia on three-vessel and tracheal view. **a** (Schematic drawing) and **b** (Ultrasound images):the left and right aortic arch on both sides of the trachea ran backward and downward. The RARCH connected with the descending aorta and the LARCH was interrupted, and diverticulum-like structure was originated from the descending aorta with its tip pointing to the left. **c** The same case on long axis of the left aortic arch view: the continuity of the distal LARCH was interrupted after sending out the LCCA and the LSCA in turn, and the left subclavian artery was twisted backward and downward, showing the sign of pulling by the tethered system. **d** The CTA 3D image of the same case: the RARCH connected with the descending aorta after sending out the right common carotid artery and the right subclavian artery. A diverticulum-like structure was originated from the descending aorta with its tip pointing to the left. The continuity of the LARCH was interrupted after sending out the LCCA and the LSCA in turn, and the LSCA was twisted backward and downward, showing the sign of pulling by the tethered system. Diverticulum: a diverticulum-like structure originated from the descending aorta with its tip pointing to the left; LARCH: left aortic arch; LCCA: left common carotid artery; LSCA: left subclavian artery; RARCH: right aortic arch; T: trachea; the distal left aortic arch was interrupted and the left subclavian artery was twisted backward and downward as shown by↑

**Fig. 6:**
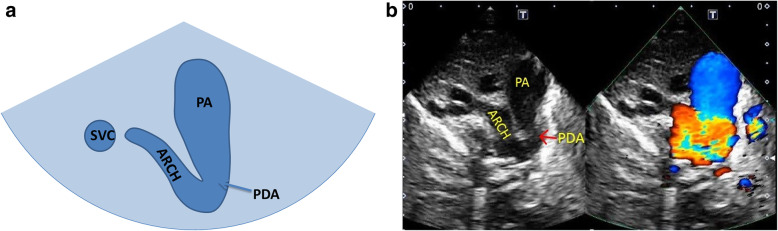
Proximal aortic atresia on three-vessel and tracheal view. **a** Schematic drawing. **b** Ultrasound images. It showed an asymmetric “V” sign. Hypoplasia of aortic arch and retrograde flow of ductus arteriosus from the pulmonary artery into the aorta could be detected. ARCH: aortic arch; PA: pulmonary artery; PDA:patent ductus arteriosus

**Fig. 7:**
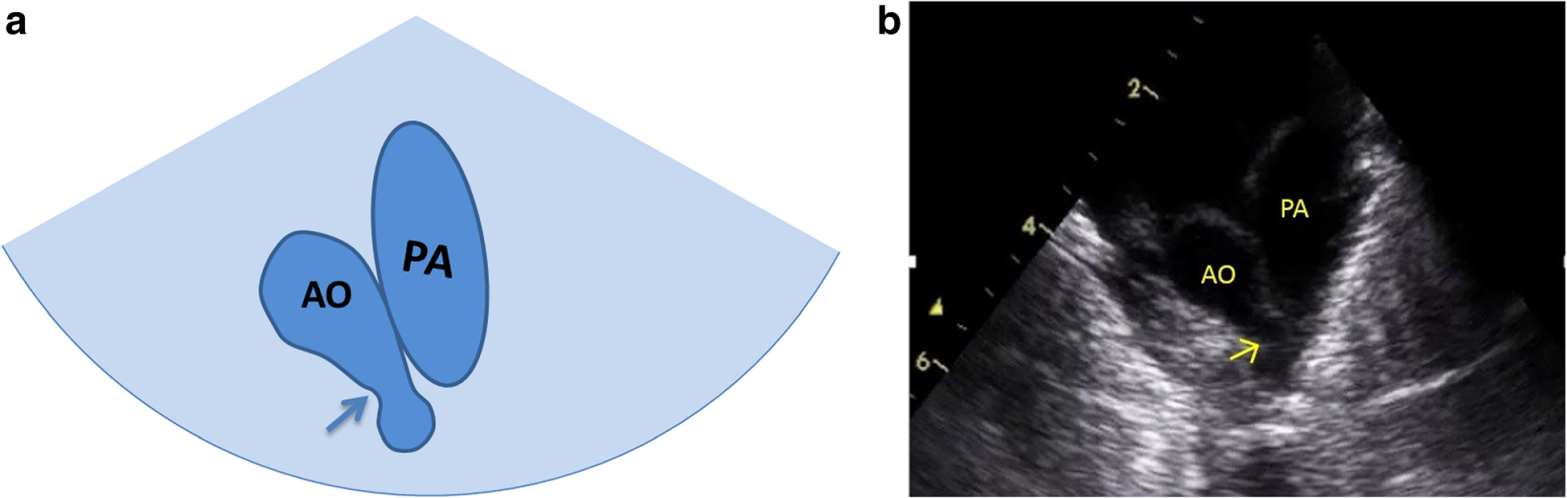
Coarctation of the aorta on three-vessel and tracheal view. **a** Schematic drawing. **b** Ultrasound images. Aortic and pulmonary artery was proportion imbalanced and the internal diameter of aorta was reduced, showing asymmetric “V” sign. AO: aorta; PA: pulmonary artery; the internal diameter of aorta reduced as shown by↑

**Fig. 8:**
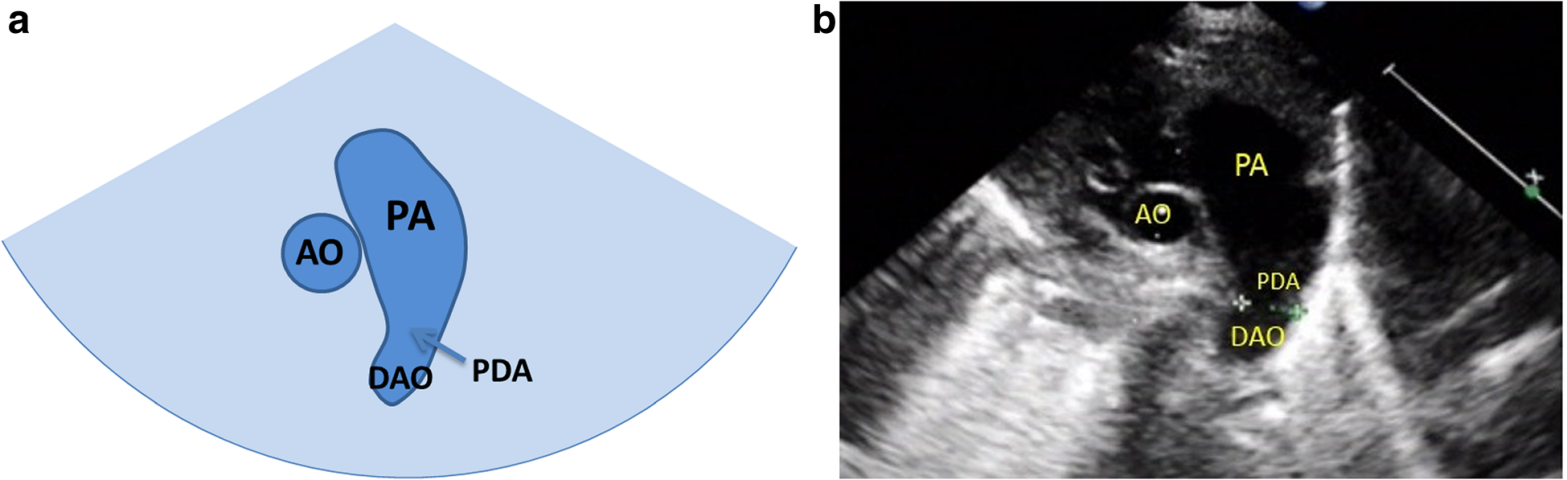
Interruption of the aortic arch on three-vessel and tracheal view. **a** Schematic drawing. **b** Ultrasound images. It showed the proportion of aortic and pulmonary artery was imbalanced, and the internal diameter of aorta was reduced. The continuity between aortic arch and descending aorta interrupted. The V-shaped structure disappeared and an “0I” shape showed. AO: aorta; DAO: descending aorta; PA: pulmonary artery; PDA: patent ductus arteriosus

1. Normal three-vessel view: superior vena cava, aorta, pulmonary artery (arranged from right to left).
2. Normal three-vessel-tracheal view: the vena cava is located on the right side of the trachea. The pulmonary artery is connected to the descending aorta through the ductus arteriosus or ductal ligament, forming a symmetrical “V” sign with the aorta (the inner diameter of the aorta and the pulmonary artery is similar), both of which are located on the left side of the trachea.
3. RAA: the distance between the aortic arch and the pulmonary artery increased. The aortic arch is located on the right side of the trachea, and the pulmonary artery is located on the left side of the trachea. The pulmonary artery is connected to the descending aorta via the arterial duct or duct ligament, forming a “U”-shaped vascular ring with the aorta. In some patients with the aberrant left subclavian artery, it could see that left subclavian artery (LSCA) originated from the descending aorta and ran to the left .
4. LAA with aberrant right subclavian artery: The aortic arch and pulmonary artery are both located on the left side of the trachea. The pulmonary artery is connected to the descending aorta through the ductus arteriosus or ductal ligament, forming a symmetrical “V” sign with the aorta. And it could see that aberrant right subclavian artery originated from the descending aorta and ran to the right along posterior of trachea, forming a “C”-shaped vascular ring.
5. DAA with both arches open: the left and right aortic arch on both sides of the trachea ran backward and downward and connected with the descending aorta, enclosing the trachea and esophagus to form an “O”-shaped vascular ring (Fig. 4).
6. DAA with left arch atresia: The left and right aortic arch on both sides of the trachea ran backward and downward. The RAA connected with the descending aorta, and the LAA was interrupted. A diverticulum-like structure originated from the descending aorta with its tip pointing to the left, forming a similar “O”-shaped vascular ring. The long axis of the left aortic arch showed that the continuity of the distal LAA was interrupted after sending out the left common carotid artery (LCCA) and LSCA in turn, and the LSCA was twisted backward and downward, showing the sign of pulling by the tethered system (Fig. 5).
7. Proximal aortic atresia: It showed an asymmetric “V” sign. Hypoplasia of aortic arch and retrograde flow of ductus arteriosus from the pulmonary artery into the aorta could be detected (Fig. 6).
8. COA: it showed the proportion of aortic and pulmonary artery was imbalanced and the internal diameter of aorta was reduced, showing an asymmetric “V” sign (Fig. 7).
9. IAA: it showed the proportion of aortic and pulmonary artery was imbalanced, and the internal diameter of aorta was reduced. The continuity between aorta and descending aorta interrupted. The V-shaped structure disappeared and an “0I” shape showed (Fig. 8).

## Discussion

A total of 6 pairs of arch arteries appeared during human embryonic development. At the 6th–8th week of the embryo, the arch arteries evolved into the basic structure of the systemic arteries. Abnormalities occurring during this period could result in a variety of aortic arch anomalies, including anomalies of size, continuity and combination of aortic arch [4].

The types of aortic arch anomalies are complex and changeable. The ultrasonic diagnosis is difficult and the misdiagnosis rate is high. Failure to detect these diseases in time is the main cause of morbidity and mortality in severe cases of infants [1]. At present, there is lack of researches on the methodology of ultrasonic screening and diagnosis of these diseases in children. This paper puts forward the views and methods of echocardiographic screening and diagnosis of these diseases after birth for the first time, which is summarized as follows:

### Views and methods of echocardiographic screening and diagnosis of aortic arch anomalies

The views researched in this study include the three-vessel and tracheal view, aortic arch short axis view, left aortic arch long axis view and aortic arch long axis continuous scan views. We found that the screening sensitivity (73.6%) and diagnostic coincidence rate (27.1%) of short axis view of aortic arch were the lowest. No abnormalities were found in 37 (37/67) cases with COA which were well developed at the proximal transverse arch near the first branch. However, compared with the traditional left aortic arch axis view, the short axis of the aortic arch increases the display of the DAA. According to the branch of the first brachiocephalic artery, we can infer the position of aortic arch, and whether there is aberrant subclavian artery. On the short axis view, the normal aortic arch shows that the first branch points to the right anterior, and the proximal bifurcation can be seen (the right brachiocephalic trunk artery sends out the right common carotid artery and right subclavian artery). When the first branch points to the right anterior and there is no bifurcation (considered as right common carotid artery), we should take LAA with the aberrant right subclavian artery into consideration. When the first branch points to the left anterior and the proximal bifurcation is visible (the left brachiocephalic trunk sends out LCCA and LSCA), it should be considered as RAA with mirror branch. When the first branch points to the left anterior and there is no bifurcation (considered as LCCA), we should take RAA with the aberrant left subclavian artery as consideration. When the short axis of the aortic arch shows a “dumbbell sign”, it indicates DAA. The screening sensitivity and diagnostic coincidence rate of left aortic arch long axis were 87.1 and 66.4% respectively, and the shape, inner diameter and continuity of LAA could be observed on this view. However, due to the limitation of angle, it is impossible to observe RAA or DAA completely. Especially when neglecting the observation of the brachiocephalic branches of aortic arch, it is inclined to misdiagnose the case of DAA. Seventeen cases of (17/20) DAA were missed by using this view alone, and the other 3 cases (3/20) were abnormal on this view because of dysplasia of LAA. We are used to concerning the observation of one side arch or dominant arch and neglecting the detection of contralateral arch and brachiocephalic artery branches. It is also one of the common causes of missed diagnosis of aortic arch anomalies, so we should pay attention in our work. The aortic arch long axis continuous scan views are modified views based on the traditional left aortic arch long axis view. It can make up for the display of RAA and DAA and improve the reveal of the aberrant subclavian artery. The screening sensitivity and diagnostic coincidence rate of aortic arch anomalies were increased to 99.3 and 95.0% respectively. These views are a series of views of aortic arch long axis sagittal and coronary dynamic scanning, which have a large amount of information, and The screening sensitivity and diagnostic coincidence rate can be significantly improved. But it demands higher requirements for the operation and observation ability of doctors. There are still some shortcomings of these views: for example, they can not directly show the spatial relationship between the aortic arch and the esophagus and trachea, so RAA and DAA could only be speculatively diagnosed. DAA with LSCA atresia is easy to be confused with mirror right arch, and all these cases need to be combined with three vessels-trachea view to complete the diagnosis.

The three-vessel and tracheal view is one of the internationally recognized important views in the echocardiographic screening of fetal congenital heart diseases [2, 3]. In this study, this view was applied to the screening and diagnosis of aortic arch anomalies in infants for the first time. The chest wall of the infant is thin. The thymus in this period has not degenerated and its volume is large, which helps to reduce the interference of air in lung and easily obtain a satisfactory three-vessel and tracheal view. During this study, except for a few cases of severe pneumonia, pneumothorax and severe thoracic deformity, the vast majority of children successfully obtained this view. The results of this study showed the screening sensitivity and diagnostic coincidence rate of this view were 99.3 and 85.7% respectively, and it had distinct advantage in the diagnosis of congenital vascular ring. In 2 cases of suspected DAA with LSCA distal atresia, although the atresia segment could not be directly displayed on ultrasound, the diagnosis of this disease was suggested according to the abnormal structural features in ultrasound (Fig. 3) consistent with those reported in the literature [5–8]. CTA and bronchoscopy were performed in the 2 cases, which confirmed the existence of tracheal compression stenosis and further supported the ultrasonic diagnosis. One case was also proved by operation. This view could easily diagnose RAA. However, because aberrant subclavian artery was located in the far sound field and had a large angle with the sound beam, it was easy to be affected by air, which could cause missed diagnosis easily. In only 2 cases with the C-shaped vascular ring of this study, 1(1/2) was missed diagnosis. Annotation: The coronal view of the descending part of aorta arch long axis with blood flow guidance can improve the display of the aberrant subclavian artery. For the patients with difficulty in directly displaying the aberrant subclavian artery, the identification of the first branch on the short axis of the aorta arch view could play an important role in speculation and diagnosis. In this group of patients with interruption, coarctation and dysplasia of aortic arch, the three-vessel and tracheal view had characteristic images. The IAA showed an”0 l” shape. Coarctation and dysplasia of aortic arch showed an asymmetric “V” sign. This view could provide important diagnostic clues of aortic arch anomalies, combining with the long axis view of aortic arch in suprasternal fossa to make an accurate diagnosis. Because of the ideal angle of this view, it can help the long-axis view of the aortic arch to distinguish the interruption or severe COA. Diagnostic skills: we first obtain the three-vessel view which can define the position of the root of the aorta and the descending aorta, then tilt the probe to the side of the head to get the three-vessel and tracheal view. During the process, the continuity of the aortic arch and the descending aorta is observed to determine whether there is any interruption or coarctation. In this study, a number of cases with controversy in the long axis of the aortic arch view were differentiated by this method, and a correct modified diagnosis was obtained. However, in the case of mild COA with a reasonable development of aortic arch, the imbalance of the aortic and pulmonary arteries in this view is inconspicuous and false negatives are prone to occur. The coarctation of the descending aorta in the lower position also can not be directly showed on this view. Therefore, we should combine this view with other views for diagnosis. This study shows that the sensitivity of screening for aortic arch abnormalities combined with four views can be increased to 100%, and the diagnostic coincidence rate can be increased to 97.9%.

### Analysis of missed and incorrectly diagnosed cases

IAA and severe COA are sometimes difficult to distinguish. In this group, 2 cases of IAA (isthmus atresia) were incorrectly diagnosed as COA and 1 case of severe COA was incorrectly diagnosed as IAA. As to these 2 cases of aortic isthmic atresia, the wall of the aortic isthmus was continuous with the descending aorta, but the lumen was atresia. The long axis of the aortic arch showed that the wall was continuous, and the tissue of the atresia segment was extremely low in echo, which was hollow lumen-like echo in two-dimensional view. The atresia segment was very short, and the false image of aortic coarctation was caused by color spillover. The three-vessel and tracheal view showed that the aortic arch was continuous with the wall of the descending aorta. The tissue of the atresia segment was very low in echo, and the two-dimensional view showed hollow lumen-like echo, presenting an asymmetric “V” sign in three-vessel and tracheal view. One case was misdiagnosed due to the identification of unopened blood flow pattern, and the other was incorrectly diagnosed by mistaking the collateral vessels for a continuous aortic arch. Encountering similar cases, especially the suspicious cases of short segment coarctation without obvious blood flow acceleration found on the long axis of the aortic arch, we can make a diagnosis by simultaneous observation on multiple views, multiple angles, two-dimension and color [9]. As the case of severe COA incorrectly diagnosed as IAA, the reason for missing diagnosis was that the coarctation segment of the aorta was lower, tortuous and slender, leading uneasily to be observed on aortic arch long axis view. Its flow was overlapped with the crassi ductus arteriosus which covered it. Due to the small diameter, tortuosity, low position of the coarctation segment and dysplasia of the aortic arch, the continuity between the aortic arch and the descending aorta was not showed on the three-vessel and tracheal view, presenting a “ol” sign, and resulted in missing diagnosis. Some IAA (atresia) were considered to be the anomaly of the most serious aortic coarctation [10]. The author ever found a case of severe coarctation of isthmus in neonatal period and developed aortic atresia 2 days later. When it is difficult to diagnose aortic arch anomalies by ultrasound or to determine the compression of vascular ring on trachea and esophagus, we can make a definite diagnosis by CTA, radiography, bronchoscopy and so on [11].

### Limitations

In this study we selected positive infant cases with aortic arch anomalies diagnosed by ultrasound and determined by CTA or/and surgery. The positive cases that could not be detected by ultrasound, the cases of loss of follow-up and the older cases with poor images were not included in this study. Incorrect diagnosis may occur in cases of mild coarctation of the distal aorta with well developed proximal aorta. The number of cases in this study was small and didn’t include all types of aortic arch anomalies. Because infant cases with aberrant subclavian artery rarely had clinical symptoms, so it was difficult to be confirmed by follow-up. In practical work, some children with congenital heart disease like this are prone to complications such as heart failure, severe pneumonia, even pneumothorax and thoracic deformities [12], which affect the clarity of the images. Prospective research needs to be carried out in the future, and the sample size should be expanded for further analysis.

## Conclusions

In general, the three-vessel and tracheal view which has high sensitivity is liable to be obtained and mastered. It can be widely applied in the ultrasonic screening and diagnosis of aortic arch anomalies in infants, and has remarkable advantages in the diagnosis of vascular ring. The combination of four views is conducive to further improve the screening sensitivity and diagnostic coincidence rate of aortic arch abnormalities.

## Data Availability

Not applicable

## Abbreviations

CTA: Computerized tomography angiography
COA: Coarctation of the aortic arch
IAA: Interruption of the aortic arch
DAA: Double aortic arch
RAA: Right aortic arch
LAA: Left aortic arch
LCCA: Left common carotid artery
LSCA: Left subclavian artery
